# PAX9 Is Involved in Periodontal Ligament Stem Cell-like Differentiation of Human-Induced Pluripotent Stem Cells by Regulating Extracellular Matrix

**DOI:** 10.3390/biomedicines10102366

**Published:** 2022-09-22

**Authors:** Risa Sugiura, Sayuri Hamano, Atsushi Tomokiyo, Daigaku Hasegawa, Shinichiro Yoshida, Hideki Sugii, Shoko Fujino, Orie Adachi, Masataka Kadowaki, Daiki Yamashita, Hidefumi Maeda

**Affiliations:** 1 Department of Endodontology and Operative Dentistry, Faculty of Dental Science, Kyushu University, Fukuoka 812-8582, Japan; 2 OBT Research Center, Faculty of Dental Science, Kyushu University, Fukuoka 812-8582, Japan; 3 Department of Endodontology, Kyushu University Hospital, Fukuoka 812-8582, Japan; 4 DDR Research Center, Faculty of Dental Science, Kyushu University, Fukuoka 812-8582, Japan

**Keywords:** induced pluripotent stem cells, periodontal ligament stem cells, extracellular matrix, transcription factor, PAX9

## Abstract

Periodontal ligament stem cells (PDLSCs) play central roles in periodontal ligament (PDL) tissue homeostasis, repair, and regeneration. Previously, we established a protocol to differentiate human-induced pluripotent stem cell-derived neural crest-like cells (iNCs) into PDLSC-like cells (iPDLSCs) using human PDL cell-derived extracellular matrix (ECM). However, it remained unclear what factors principally regulate the differentiation of iNCs into iPDLSCs. In this study, we aimed to identify the transcription factor regulating production of human PDL cell-derived ECM, which is responsible for the generation of iPDLSCs. We cultured iNCs on ECMs of two human PDL cell lines (HPDLC-3S and HPDLC-3U) and of human dermal fibroblasts (HDF). iNCs cultured on HPDLC-3U demonstrated higher iPDLSC-associated gene expression and mesenchymal differentiation capacity than cells cultured on HDF or HPDLC-3S. The transcription factor *PAX9* was highly expressed in HPDLC-3U compared with HDF and HPDLC-3S. iNCs cultured on siPAX9-transfected HPDLC-3U displayed downregulation of iPDLSC-associated marker expression and adipocytic differentiation capacity relative to controls. Our findings suggest that PAX9 is one of the transcription factors regulating ECM production in human PDL cells, which is responsible for the differentiation of iNCs into iPDLSCs.

## 1. Introduction 

Periodontal ligament (PDL) is a highly specialized fibrous connective tissue that plays important roles in anchoring the tooth to the socket bone and regulating proper tooth homeostasis, repair, and nutrition [1]. One of the primary causes of PDL destruction is an advanced inflammatory disease of tissues surrounding the tooth structure, periodontitis. Once PDL is lost by the progression of periodontitis, the socket bone becomes detached from the tooth root and can no longer provide support for the tooth. PDL destruction can ultimately result in tooth loss; therefore, researchers have tried to repair and regenerate injured PDL tissue with the aim of preserving teeth with severe periodontitis.

PDL consists of a heterogeneous cell population, including fibroblasts, osteoblasts, cementoblasts, endothelial cells, and epithelial cell rests of Malassez [2]. Additionally, PDL contains a subpopulation of mesenchymal stem cells. Seo et al. isolated somatic mesenchymal stem cells from PDL tissues (periodontal ligament stem cells, PDLSCs) of surgically extracted human third molars, which demonstrated multipotency, high proliferative capacity, and the ability to regenerate bone, cementum, and PDL tissue [3]. PDLSCs also exhibit immunomodulatory abilities via suppression of T cell, B cell, and dendritic cell activities, and promotion of Treg generation [4,5,6,7]. In addition, autologous human PDLSC transplantation can reduce the clinical attachment level, probing depth, and defect area, and increase bone density [8]. Therefore, PDLSCs are crucial for the repair and regeneration of PDL tissue. However, the stem cell population in PDL tissue is extremely rare, as with many other tissues, and tooth extraction is required to obtain PDLSCs. Acquisition of many PDLSCs is required for clinical application to widespread PDL destruction, which remains quite difficult. 

In 2006, Takahashi and Yamanaka reprogramed somatic cells by forced expression of the transcription factors *Oct4*, *Klf4*, *Sox2*, and *c-Myc* [9]. The reprogramed cells acquired properties like those of embryonic stem cells (ESCs) including cell morphology, pluripotency, marker expression, and high proliferative capacity. These cells were named induced pluripotent stem cells (iPSCs) based on their characteristics. Because they can be generated from individual somatic cells, iPSCs have the advantage of limited immune rejection upon autologous transplantation. Moreover, these cells overcome ethical issues associated with isolating human ESCs from blastocysts. Therefore, iPSCs have revolutionized the field of personalized medical research, including in vitro disease modeling, drug screening, and regenerative cell therapy [10,11,12,13].

Various types of somatic cells, such as neural cells [14], kidney cells [15], cardiomyocytes [16], retinal pigment epithelial cells [17], intestinal epithelial cells [18], and blood cells [19] have been generated from iPSCs. We also developed a protocol to induce the differentiation of human iPSCs into PDLSC-like cells (iPDLSCs) exhibiting high expression of genes strongly expressed in PDLSCs, including Type I collagen (*COL1*), Fiblillin-1 (*FBN1*), Osteoprotegerin (*OPG*), and PDL-associated protein-1 (*POSTN*) [20]. iPDLSCs also display high proliferative potential, mesenchymal stem cell-related marker expression, and mesenchymal-lineage (osteoblasts and adipocytes) differentiation potential. Therefore, iPDLSCs closely resemble PDLSCs and could become a promising cell source for regenerative cell therapy of PDL tissue. 

An extracellular matrix (ECM), consisting of a large and varied group of macromolecules and their regulatory factors, is involved in tissue development, homeostasis, repair, and regeneration by regulating cell differentiation, migration, proliferation, apoptosis, and morphology [21,22]. iPDLSCs are generated from iPSC-derived neural crest cells (iNCs) by culturing these cells on ECM derived from human PDL cells [20]. To improve the efficiency of this process, it is necessary to identify ECM components that more effectively differentiate iNCs into iPDLSCs. However, PDL cells can produce various types of ECM, such as collagen, fibronectin, proteoglycan, osteopontin, bone sialoprotein, and osteonectin [23,24,25,26]. Moreover, the composition of ECM varies depending on factors such as sex, age, and the type of tissue [27]. ECM derived from human PDL cells is heterogeneous and its function is wide-ranging and complicated; therefore, it is difficult to isolate one crucial component for inducing iPDLSCs from iNCs.

Transcription factors regulate the expression of various genes by directly binding specific DNA sequences located in gene promoters and distal regulatory elements [28]; therefore, they are considered to engage a variety of important biological responses. Previous publications demonstrated that various transcription factors, such as SMAD3, Sp1, STAT5, Egr-1, and Nrf2 have the ability to interact with the promoters of ECM-related genes and regulate their expression [29,30,31,32,33]. Additionally, AP1 [34] and Mkx [35] can reportedly act as regulators of ECM-related gene expression in PDL cells. However, the transcription factors that regulate ECM production in human PDL cells—an important factor for differentiation of iNCs into iPDLSCs—have not been clarified. Given these hallmarks, we aimed to isolate the transcription factor responsible for generation of iPDLSCs by gene expression analysis and gene ontology (GO) enrichment analysis techniques using human PDL cells and dermal fibroblasts.

## 2. Materials and Methods

### 2.1. Cell Culture

Human dermal fibroblasts (HDFs) and human iPSCs were obtained from RIKEN (Ibaraki, Japan; HPS No. RCB0156 and No. 0063). Human iPSCs were cultured on mouse embryonic fibroblasts (MEFs; ReproCELL, Kanagawa, Japan) and maintained in primate ESC medium (ReproCELL) containing 5 ng/mL human recombinant basic fibroblast growth factor (b-FGF; ReproCELL) at 37 °C in a humidified atmosphere of 5% CO_2_ and 95% air. Two human PDL cell populations were isolated from the third molar of a healthy 25-year-old female patient (HPDLC-3U) and 23-year-old male patient (HPDLC-3S), who visited Kyushu University for extraction, as described previously [36]. Informed consent was obtained from both tissue donors according to the guidelines of the Research Ethics Committee of Kyushu University Certified Institutional Review Board for Clinical Trials. HDF and HPDLCs were maintained in alpha-Minimum Essential Medium (α-MEM; Gibco-BRL, Grand Island, NY, USA) supplemented with 50 U/µL penicillin and 50 µg/mL streptomycin (Wako, Osaka, Japan) containing 10% fetal bovine serum (FBS; Sigma-Aldrich, St. Louis, MO, USA) (10% FBS/α-MEM) at 37 °C in a humidified atmosphere of 5% CO_2_ and 95% air. Cells from passages 5 through 7 were used in this study. All procedures were performed following the rules of the Declaration of Helsinki and performed in compliance with the Research Ethics Committee of Kyushu University Certified Institutional Review Board for Clinical Trials. (Approval No. 2-115, 10 August 2020). We confirm that all experiments were performed in according to relevant guidelines and regulations. 

### 2.2. Quantitative Reverse Transcription Polymerase Chain Reaction

Total RNA was isolated from each cells using TRIzol Reagent (Invitrogen, Carlsbad, CA, USA), and first-strand cDNA was removed from 500 ng of total RNA with an ExScript RT Reagent kit (Takara Bio, Kusatsu, Japan) according to the manufacturer’s instructions. Quantitative RT-PCR assays were performed using a KAPA SYBR Fast qPCR kit (Nippon Genetics, Tokyo, Japan) in a Thermal Cycler Dice Real-Time System (Takara Bio) according to the manufacturer’s instructions. as described previously [20]. Specific primer sequences, GenBank ID, product sizes, and annealing temperatures for each gene are listed in Table 1. β-actin was used as an internal control in all quantitative RT-PCR. The expression levels of target genes were determined using the 2^−ΔΔCt^ method. Primer sequences were designed using the GenBank database (NCBI), and primer specificity was confirmed by GenBank BLAST searches. 

### 2.3. Differentiation of iPSC-Derived Neural Crest Cells (iNCs) Cultured on ECM Derived from HDF, HPDLC-3S, or HPDLC-3U

Human iPSCs were first differentiated into iNCs according to a previous report [37]. Briefly, separated human iPSCs were cultured on Matrigel-coated dishes with MEF-conditioned medium supplemented with 10 mM Rho-associated protein kinase inhibitor (Enzo Life Sciences, Farmingdale, NY, USA) and 10 ng/mL b-FGF. After the cultures reached subconfluence, cells were cultured in neural crest cell differentiation medium composed of KnockOut DMEM (Gibco-BRL) supplemented with 15% KnockOut Serum Replacement, 1% L-glutamine, 1% non-essential amino acids, 50 U/mL penicillin, 50 mg/mL streptomycin, 0.1% β-mercaptoethanol (Sigma-Aldrich), 500 ng/mL Noggin (PeproTech, Inc., Rocky Hill, NJ, USA), and 10 mM SB431542 (Tocris, Minneapolis, MN, USA) according to culture periods. Following of 10 days incubation, cells were subjected to magnetic-activated cell sorting (Miltenyi Biotec, Bergisch Gladbach, Germany) using a bead-conjugated p75NTR antibody (Miltenyi Biotec) for the isolation of iNCs. HDF, HPDLC-3S, and HPDLC-3U cells were separately cultured in 10% FBS/α-MEM until reaching confluency and then detached with 2% EDTA (Nacalai Tesque, Kyoto, Japan) in phosphate-buffered saline (PBS) to obtain dishes coated with ECM. iNCs were seeded on these ECM-coated dishes and further cultured for 2 weeks. Following culture, iNCs exposed to ECM derived from HDF, HPDLC-3S, and HPDLC-3U were named iNC-HDF, iNC-3S, and iNC-3U, respectively.

### 2.4. Cap Analysis Gene Expression and Gene Ontology Enrichment Analysis

Cap analysis of gene expression (CAGE) allows genome-wide analysis of gene transcription start sites and quantitative study of RNA transcribed from them by DNAFORM (Yokohama, Kanagawa, Japan). In brief, RNA quality was assessed with a Bioanalyzer (Agilent, Santa Clara, CA, USA) to ensure that the RNA integrity number was over 7.0, and A260/280 and 260/230 ratios were over 1.7. First-strand cDNAs were transcribed to the 5ʹ end of capped RNAs and attached to CAGE “barcode” tags, which upon sequencing were mapped to the mouse mm9 genomes using BWA software (v.0.5.9, SourceForge Headquarters, San Diego, CA, USA) after discarding ribosomal or non-A/C/G/T base-containing RNAs. For tag clustering, CAGE-tag 5ʹ coordinates were input for CAGEr clustering using 20 bases as a maximal allowed distance between two neighboring tags and a minimum counts per million (CPM) value of 2 [38]. GO enrichment analysis was also performed using BWA and Gene Set Enrichment Analysis (GSEA) software (Broad Institute, Cambridge, MA, USA) [39,40]. 

### 2.5. Small Interfering RNA Transfection

Small interfering RNA (siRNA) for human *PAX9* (MISSION siRNA, SASI_Hs02_00341076; Sigma-Aldrich) or human control siRNA (MISSION siRNA Universal Negative Control #1, SIC-001-10; Sigma-Aldrich) were introduced into HPDLC-3U using Lipofectamine RNAiMAX (Invitrogen) according to the manufacturer’s instructions, as described previously [41]. Briefly, HPDLC-3U cells were seeded in 24-well plates (Becton Dickinson Labware, Lincoln Park, NJ, USA) at a density of 1 × 10^4^ cells per well in Opti-MEM (Invitrogen) containing 10% FBS. After they reached 50–70% confluency, siRNA was transduced. The siRNA-lipid complex was prepared by mixing 10 pmol siRNA and 1.5 µL of Lipofectamine RNAiMAX in 50 µL Opti-MEM. The complex was incubated for 5 min at room temperature, then added to cells and the mixture was incubated for 48 h. HPDLC-3U transfected with control siRNA and PAX9 siRNA were designated 3U + siCont and 3U + siPAX9, respectively. Untransfected HPDLC-3U was designated Unt-3U.

### 2.6. Differentiation of iPSC-Derived Neural Crest Cells (iNCs) Cultured on ECM Derived from Unt-3U, 3U + siCont, and 3U + siPAX9

Unt-3U, 3U + siCont, and 3U + siPAX9 were cultured in 10% FBS/α-MEM until reaching confluency. Subsequently, cells were detached with 2% EDTA in PBS and then iNCs were seeded on these ECM-coated dishes and cultured for 2 weeks. iNCs exposed to ECM derived from Unt-3U, 3U + siCont, and 3U + siPAX9 were designated iNC-Unt, iNC-siCont, and iNC-siPAX9, respectively.

### 2.7. Immunofluorescence Staining

iNC-Unt, iNC-siCont, and iNC-siPAX9 were fixed with 4% paraformaldehyde (Nacalai Tesque, Kyoto, Japan) and 0.5% dimethyl sulfoxide (Nacalai Tesque) in PBS for 30 min. We used 2% bovine serum in PBS to block cells for 1 h, then cells were incubated with rabbit polyclonal anti-human OPG (5 µg/mL; Abcam, Cambridge, UK), rabbit polyclonal anti-human POSTN antibody (1:100 dilution; Sigma-Aldrich), or rabbit polyclonal IgG isotype control antibody (Cell Signaling Technology, Beverly, MA) overnight at 4 °C. The following day, cells were washed three times with PBS and incubated with an Alexa Fluor 647-conjugated chicken anti-rabbit antibody (1:250 dilution; Invitrogen). After 1 h, the cells were washed three times with PBS and counterstained with DAPI (Nacalai Tesque). Stained cells were imaged and analyzed using a Biozero digital microscope (Keyence Corporation, Osaka, Japan). 

### 2.8. Flow Cytometric Analysis

iNC-Unt, iNC-siCont, and iNC-siPAX9 were detached with Accutase (ReproCELL) and adjusted to a density of 5 × 10^5^ cells/tube. Next, cells were incubated with R-phycoerythrin-conjugated mouse anti-human CD 34, CD45, CD90, CD105, CD166 (BioLegend, San Diego, CA, USA), IgG1, or IgG2a (iCyte, Tokyo, Japan) for 45 min at 4 °C. Cells were then washed with flow cytometry buffer (R&D Systems, Minneapolis, MN, USA) and percentages of positive cells were measured by flow cytometry (EC800 Cell Analyzer; Sony, Tokyo, Japan). Data were analyzed using Eclipse software (Sony, Tokyo, Japan). 

### 2.9. Proliferation Assay

iNC-Unt, iNC-siCont, and iNC-siPAX9 were seeded at a density of 3 × 10^3^ cells/well into wells of 48-well plates and incubated for up to 7 days. Subsequently, their proliferation was examined using a cell proliferation assay kit (Takara Bio, Shiga, Japan) on 0, 1, 2, 3, 5, and 7 days. After incubation, 25 µL of the WST-1 kit reagent was added to the culture medium of each well. Following 1 h of treatment, 100 µL of supernatant was collected from each well, and the optical density at 450 nm of each well was measured with an iMark microplate reader (Bio-Rad Laboratories, Hercules, CA, USA).

### 2.10. Osteoblastic Differentiation

iNC-HDF, iNC-3S, iNC-3U, iNC-Unt, iNC-siCont, and iNC-siPAX9 were seeded at a density of 2 × 10^4^ cells into wells of a 24-well plate and cultured in 10% FBS/α-MEM (control medium; CM). After reaching confluence, the culture medium was changed to an osteoblastic differentiation medium (ODM) composed of CM supplemented with 50 µg/mL ascorbic acid (Nacalai Tesque) and 2 mM β-glycerophosphate (Sigma Aldrich). As a control, cells were cultured in CM. After 3 weeks of culture, cells were fixed with 10% formalin (Wako) for 1 h, washed with distilled water, and stained with 0.3% Alizarin Red S (Invitrogen), as described previously [16]. Images of stained cell were captured with a Keyence BZ-9000 microscope (Osaka, Japan). Nine fields were randomly chosen for quantification of Alizarin Red S-positive area. Measurements were performed using BZ-X Analyzer Software (Keyence, Osaka, Japan).

### 2.11. Adipocytic Differentiation

iNC-HDF, iNC-3S, iNC-3U, iNC-Unt, iNC-siCont, and iNC-siPAX9 were seeded at a density of 2 × 10^4^ cells into wells of a 24-well plate and cultured in CM. After reaching confluence, the culture medium was changed to an adipocytic differentiation medium (ADM) composed of CM supplemented with 1% L-glutamine, 0.1 mM L-ascorbic acid (Wako), 1 mM Sodium Pyruvate Solution (100×, Nacalai Tesque), 10 µM hydroxyethyl-piperazinyl ethanesulfonic acid (Nacalai tesque), 60 mM indomethacin (Sigma-Aldrich), and 10^−7^ M dexamethasone (Merck Millipore, Darmstadt, Germany). As a control, cells were cultured in CM. After 4 weeks, cells were fixed with 10% formalin, washed with distilled water, and stained with 0.3% Oil Red O (Invitrogen) for lipid detection as described previously [16]. Images of stained cell were captured with a Keyence BZ-9000 microscope. Nine fields were randomly chosen for quantification of the number of Oil Red O-positive cells. 

### 2.12. Statistical Analysis

All values are expressed as the mean ± standard deviation. Statistical analysis was performed using one-way ANOVA, followed by the Bonferroni method for comparisons of three or more groups. *p* values of < 0.05 were considered statistically significant.

## 3. Results

### 3.1. Periodontal Ligament Stem Cell Characteristics of iNC-HDF, iNC-3S, and iNC-3U

**Figure 1:**
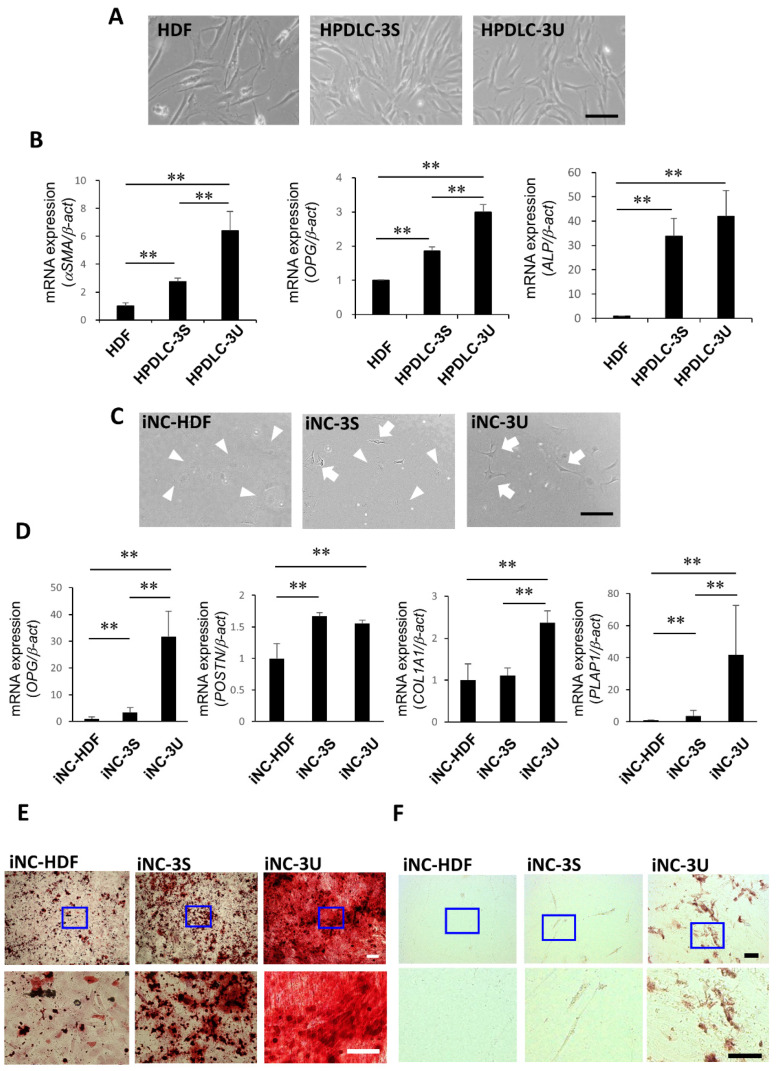
Comparison of PDLSC-like characteristics between iNC-HDF, iNC-3S, and iNC-3U (**A**) Representative phase-contrast microscopic images of HDF, HPDLC-3S, and HPDLC-3U. Bar = 100 μm. (**B**) Quantitative real time RT-PCR analysis of PDL-related genes *α-SMA*, *OPG*, and *ALP*, in HDF, HPDLC-3S, and HPDLC-3U. β-act was used as an internal standard. Data portrayed are the mean ± standard deviation (*n* = 4). ** *p* < 0.01. (**C**) Representative phase-contrast microscopic images of iNC-HDF, iNC-3S, and iNC-3U. White arrowheads indicate cells with rounded shapes and white arrows indicate cells with spindle shapes. Bar = 100 µm. (**D**) Quantitative real-time RT-PCR analysis of iPDLSC-associated genes *OPG*, *POSTN*, *COL1A1*, and *PLAP1* in iNC-HDF, iNC-3S, and iNC-3U. *β-act* was used as an internal standard. Data portrayed are the mean ± standard deviation (*n* = 4). ** *p* < 0.01. (**E**) Representative Arizarn Red S-staining images of iNC-HDF, iNC-3S, and iNC-3U after 3 weeks of culture in ODM. Upper panels portrayed the lower magnification images of iNC-HDF, iNC-3S, and iNC-3U. Lower panels portrayed the higher magnification images of blue boxes in the upper panels. Bars = 100 µm. (**F**) Representative Oil red O-staining images of iNC-HDF, iNC-3S, and iNC-3U after 4 weeks of culture in ADM. Upper panels portrayed the lower magnification images of iNC-HDF, iNC-3S, and iNC-3U. Lower panels portrayed the higher magnification images of blue boxes in the upper panels. Bars = 100 μm. HDF, human dermal fibroblasts; HDPLC, human periodontal ligament cell; iNC, neural crest-like cells derived from human induced pluripotent stem cells; iPDLSC, periodontal ligament stem cell-like cells derived from iNC; ODM, osteoblastic differentiation medium; ADM, adipocytic differentiation medium.

HDFs, HPDLC-3S cells, and HPDLC-3U cells demonstrated typical fibroblastic morphologies, namely plump spindle or stellate shapes with centrally placed round nuclei (Figure 1A). HPDLC-3S and HPDLC-3U cells highly expressed PDL-related genes *αSMA*, *OPG*, and *ALP* compared with HDFs (Figure 1B). These results indicated that HPDLC-3U, which is a heterogeneous cell population, contained more fibroblasts expressing PDL-related genes than HDF and HPDLC-3S.

iNC-HDF mainly exhibited rounded shapes, while iNC-3S included cells with round or spindle shapes (Figure 1C). iNC-3U was mainly comprised of spindle-shaped cells (Figure 1C). iNC-3U and iNC-3S displayed higher expression of iPDLSC-associated genes *OPG*, *POSTN*, *COL1A1*, and *PLAP1* compared with HDF (Figure 1D). Additionally, expression of *OPG*, *COL1A1*, and *PLAP1* was upregulated in iNC-3U compared with iNC-3S (Figure 1D). After osteoblastic induction, iNC-HDF formed a small number of Arizarin Red S-positive mineralized deposits, while iNC-3S generated more mineralized deposits than iNC-HDF (Figure 1E). iNC-3U formed large numbers of mineralized deposits compared with iNC-HDF and iNC-3S (Figure 1E). Following adipocytic induction, iNC-HDF formed no Oil Red O-positive lipid droplets and iNC-3S generated only a small number of lipid droplets (Figure 1F). In contrast, iNC-3U formed large numbers of lipid droplets compared with iNC-HDF and iNC-3S (Figure 1F). These results indicated that ECM derived from HPDLC-3U had higher induction ability to PDLSC compared with ECM derived from HPDLC-3S and HDF.

### 3.2. Identification of Transcription Factor PAX9 Responsible for the Induction of iNCs to iPDLSCs

**Figure 2:**
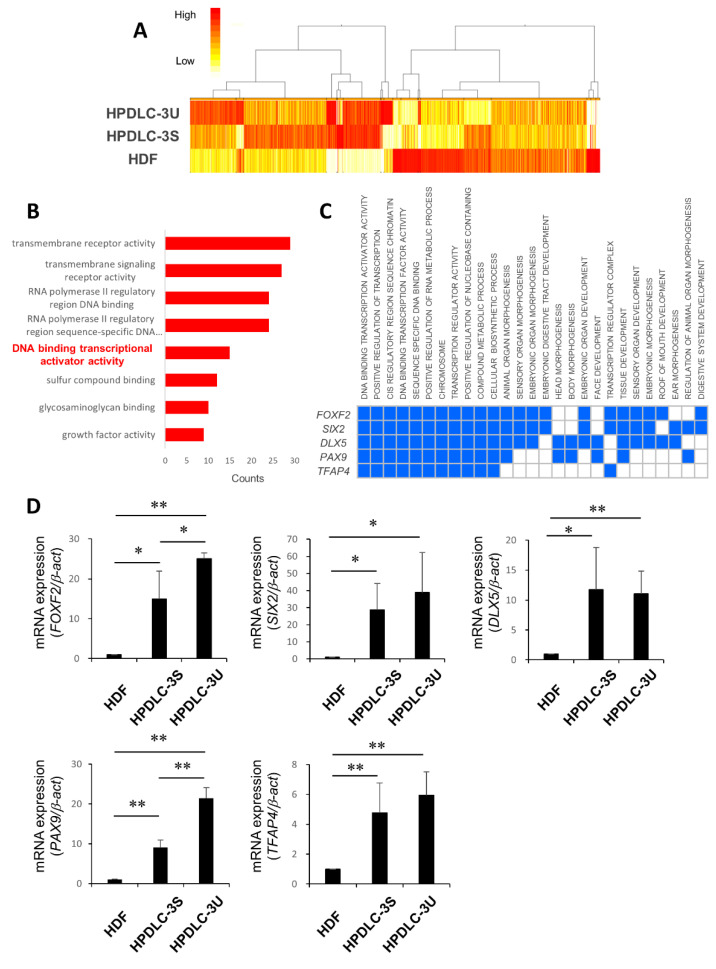
Identification of transcription factor PAX9 responsible for the induction of iNCs to iPDLSCs. (**A**) Heat map and clustering based on hierarchy for gene expression data from CAGE datasets in HDF, HPDLC-3S, and HPDLC-3U. Upregulated genes are represented in red and downregulated genes are represented in yellow. (**B**) GO analysis by BWA software based on upregulated genes in HPDLC-3S compared with HDF in the molecular function group. Data portray the top eight significant terms according to enrichment factor. The term “DNA binding transcriptional activator activity” included 15 genes. (**C**) GO analysis by GSEA software for *FOXF2*, *SIX2*, *DLX5*, *PAX9*, and *TFAP4*. Blue color means the GO term in which each gene is involved. (**D**) Quantitative real time RT-PCR analysis of *FOXF2*, *SIX2*, *DLX5*, *PAX9*, and *TFAP4* in HDF, HPDLC-3S, and HPDLC-3U. *β-act* was used as an internal standard. Data are portrayed as the mean ± standard deviation (*n* = 4). * *p* < 0.05, ** *p* < 0.01. CAGE, cap analysis of gene expression; GO, gene ontology; GSEA, gene set enrichment analysis.

Expression levels of iPDLSC-related genes and their ability to differentiate into osteoblasts and adipocytes varied between iNC-HDF, iNC-3S, and iNC-3U. Therefore, we performed CAGE to compare gene expression among HDF, HPDLS-3S, and HPDLC-3U. A total of 13728 genes were screened out (Figure 2A), of which, 1986 were upregulated in HPDLC-3S by >2-fold relative to HDF. GO analysis indicate that the term “DNA binding transcriptional activator activity” was enriched in HPDLC-3S compared with HDF, and 15 genes were included in this term (Figure 2B). Among them, expression of five genes (*FOXF2*, *SIX2*, *DLX5*, *PAX9*, and *TFAP4*) was upregulated in HPDLC-3U relative to HPDLC-3S. GSEA confirmed that these genes played various roles not only in regulation of transcription, but also compound metabolic processes, cellular biosynthetic processes, and animal organ morphogenesis (Figure 2C). Quantitative RT-PCR analysis performed to investigate their expression in HDF, HPDLC-3S, and HPDLC-3U indicated that expression of all fives genes was significantly higher in HPDLC-3S than in HDF (Figure 2D). Expression of these genes was also significantly higher in HPDLC-3U than in HDF; however, expression of *SIX2*, *DLX5*, and *TFAP4* demonstrated no difference between HPDLC-3S and HPDLC-3U (Figure 2D). Moreover, *FOXF2* and *PAX9* expression was significantly higher in HPDLC-3U than in HPDLC-3S (Figure 2D). Further comparison of their expression levels in HPDLC-3S and HPDLC-3U revealed that the rate of increase was higher for *PAX9* than *FOXF2* (Figure 2D). Because of these analyses, we focused on *PAX9* in this study.

### 3.3. PAX9 Gene Expression in Unt-3U, 3U + siCont, and 3U + siPAX9

**Figure 3:**
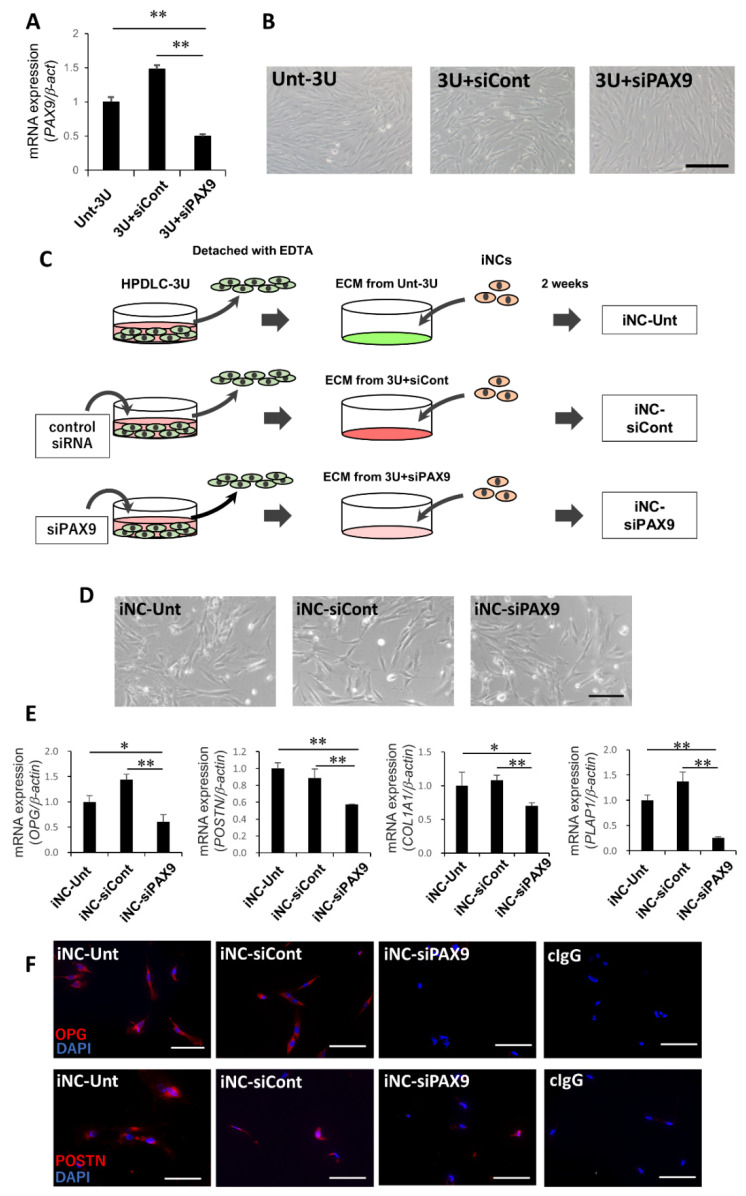
The downregulation of gene and protein expression of iPDLSC-associated markers in iNC-siPAX9. (**A**) Quantitative real-time RT-PCR analysis of PAX9 in Unt-3U, 3U + siCont, and 3U + siPAX9. β-act was used as an internal standard. Data are portrayed as the mean ± standard deviation (*n* = 4). ** *p* < 0.01. (**B**) Representative phase-contrast microscopic images of Unt-3U, 3U + siCont, and 3U + siPAX9. Bar = 200 μm. Unt-3U, untransfected HPDLC-3U; 3U + siCont, control siRNA-transfected HPDLC-3U; 3U + siPAX9, siPAX9-transfected HPDLC-3U. (**C**) Schema of generation methods for iNC-Unt, iNC-siCont, and iNC-siPAX9. iNCs were cultured for 2 weeks on ECM derived from Unt-3U, 3U + siCont, or 3U + siPAX9. (**D**) Representative phase-contrast microscopic images of iNC-Unt, iNC-siCont, and iNC-siPAX9. Bar = 200 µm. (**E**) Quantitative real time RT-PCR analysis of iPDLSC-associated genes *OPG*, *POSTN*, *COL1A1*, and *PLAP1* in iNC-Unt, iNC-siCont, and iNC-siPAX9. *β-act* was used as an internal standard. Data are portrayed as the mean ± standard deviation (*n* = 4). * *p* < 0.05, ** *p* < 0.01. (**F**) Immunocytochemical staining images of OPG and POSTN in iNC-Unt, iNC-siCont, and iNC-siPAX9. iNC-Unt were also stained with control IgG. The positive reaction was represented by red color. Nuclei were counterstained by DAPI (blue). Bars = 100 µm. iNC-Unt; iNCs cultured on ECM derived from Unt-3U, iNC-siCont; iNCs cultured on ECM derived from 3U + siCont, iNC-siPAX9; iNCs cultured on ECM derived from 3U + siPAX9. ECM, extracellular matrix; iNC, induced pluripotent stem cell-derived neural crest-like cells.

To evaluate the effect of *PAX9* on the ability of ECM derived from HPDLC-3U to induce differentiation of iNCs into iPDLSCs, we transfected si-PAX9 into HPDLC-3U to downregulate PAX9 expression. *PAX9* expression was significantly decreased in 3U-siPAX9 compared with Unt-3U and 3U-siCont (Figure 3A). However, Unt-3U, 3U + siCont, and 3U + siPAX9 revealed typical fibroblastic shapes and almost identical morphologies (Figure 3B).

### 3.4. iPDLSC-Associated Marker Expression in iNC-Unt, iNC-siCont, and iNC-siPAX9

Next, we generated iNC-Unt, iNC-siCont, and iNC-siPAX9 by culturing iNCs on Unt-3U, 3U + siCont, and 3U + siPAX9 for 2 weeks, respectively (Figure 3C). iNC-Unt, iNC-siCont, and iNC-siPAX9 exhibited spindle shapes and did not include cells exhibiting rounded shapes (Figure 3D). Expression levels of *OPG*, *POSTN*, *COL1A1*, and *PLAP1* were almost identical between iNC-Unt and iNC-siCont (Figure 3E). However, expression of these genes was significantly downregulated in iNC-siPAX9 compared with iNC-Unt and iNC-siCont (Figure 3E). Moreover, many cells were positive for OPG and POSTN in iNC-Unt and iNC-siCont, while few cells demonstrated positive reactions in iNC-siPAX9 (Figure 3F). No staining was observed in iNC-Unt stained with control IgG (Figure 3F). These results suggested that PAX9 was involved in the differentiation of iNC into iPDLSC.

### 3.5. Proliferation and Cell Surface Marker Expression in iNC-Unt, iNC-siCont, and iNC-siPAX9

**Figure 4:**
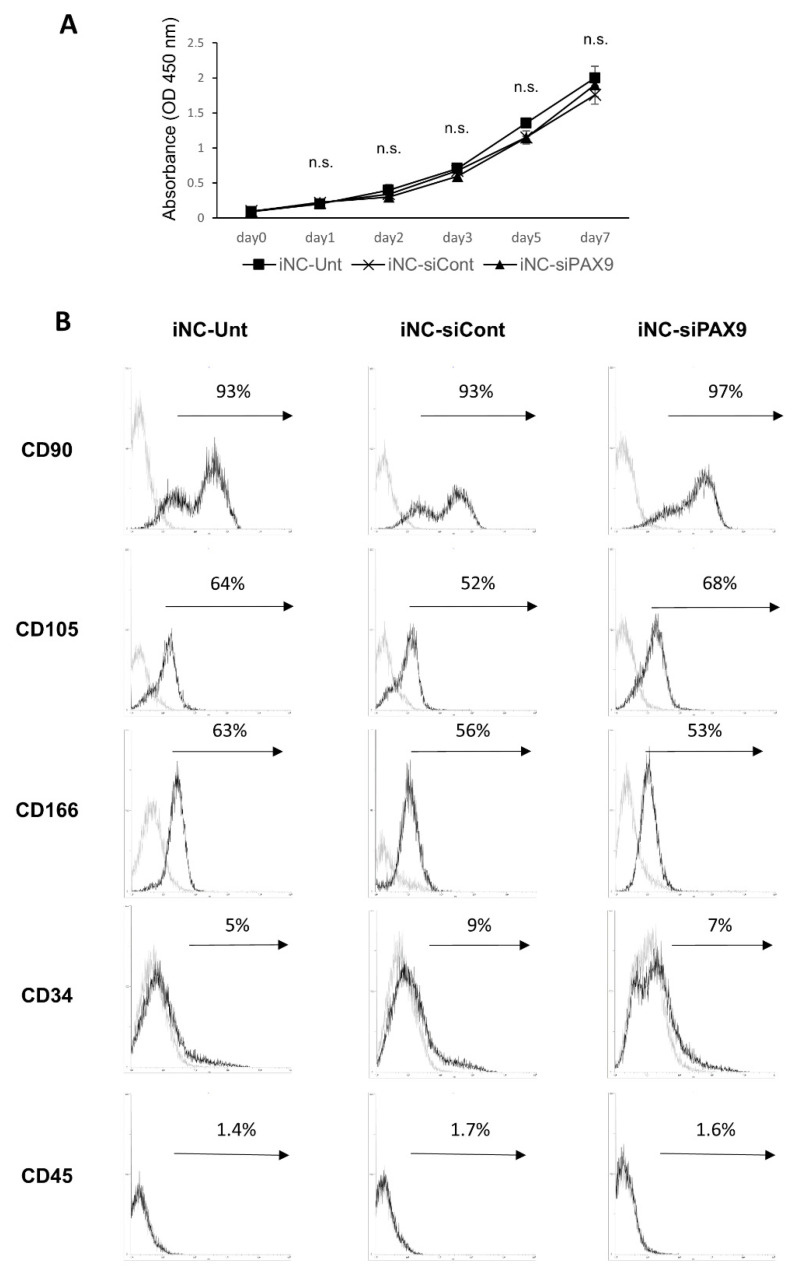
No significant difference in proliferative capacity and cell surface antigen marker expression between iNC-Unt, iNC-siCont, and iNC-siPAX9. (**A**)Proliferation of iNC-Unt, iNC-siCont, and iNC-siPAX9 determined by WST-1 assay. (**B**) Flow cytometric analysis of MSC (CD90, CD105, and CD166)- and hematopoietic cell (CD34 and CD45)-associated surface marker expression in iNC-Unt, iNC-siCont, and iNC-siPAX9. Histograms depicting fluorescently labelled cells with MSC- and hematopoietic-associated markers (black lines), and isotype-matched control (gray lines). n.s., not significant; iNC, induced pluripotent stem cell-derived neural crest-like cells; MSC, mesenchymal stem cell.

iNC-Unt, iNC-siCont, and iNC-siPAX9 exhibited a time-dependent increase in proliferation (Figure 4A). iNC-Unt exhibited higher levels of proliferation than iNC-siCont and iNC-siPAX9 on days 2, 3, 5, and 7; however, there was no statistically significant difference in proliferation between iNC-Unt, iNC-siCont, or iNC-siPAX9 from days 1–7 (Figure 4A). Cells highly expressed the MSC-associated surface markers CD90, CD105, and CD166, and slightly expressed the hematopoietic cell-associated markers CD34 and CD45 (Figure 4B). Moreover, expression levels of MSC- and hematopoietic-associated markers were almost identical between iNC-Unt, iNC-siCont, and iNC-siPAX9 (Figure 4B). These data indicated that PAX9 did not affect proliferative potential or expression of surface antigen markers in differentiation into iPDLSCs.

### 3.6. Osteoblastic Differentiation of iNC-Unt, iNC-siCont, and iNC-siPAX9

**Figure 5:**
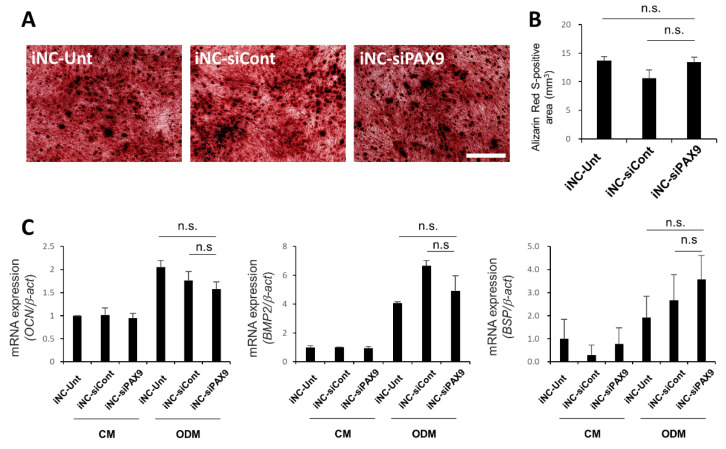
No significant difference in osteoblastic differentiation between iNC-Unt, iNC-siCont, and iNC-siPAX9. (**A**) Representative Alizarin Red S-staining in iNC-Unt, iNC-siCont, and iNC-siPAX9 after 3 weeks of culture in ODM. Bar = 200 µm. (**B**) The area of Alizarin Red S-positive staining in iNC-Unt, iNC-siCont, and iNC-siPAX9 cultured in ODM for 3 weeks. (**C**) Quantitative real time RT-PCR analysis of osteoblast-associated marker genes *OCN*, *BMP2*, and *BSP* in iNC-Unt, iNC-siCont, and iNC-siPAX9 after 3 weeks of culture in CM or ODM. *β-act* was used as an internal standard. Data are portrayed as the mean ± standard deviation (*n* = 4). CM; control medium (10% FBS/α-MEM). CM, conditioned medium; iNC, induced pluripotent stem cell-derived neural crest-like cells; ODM, osteoblastic differentiation medium. n.s.: non significant.

After 3 weeks of culture in ODM, iNC-Unt, iNC-siCont, and iNC-siPAX9 formed large numbers of Alizarin Red S-positive mineralized deposits (Figure 5A). Indeed, there was no statistically significant difference in Alizarin Red S-positive area between cultures (Figure 5B). In contrast, no deposits were observed in iNC-Unt, iNC-siCont, and iNC-siPAX9 treated with CM (Figure S1A). Expression of osteoblast-related markers *OCN*, *BMP2*, and *BSP* was upregulated in iNC-Unt, iNC-siCont, and iNC-siPAX9 cultured in DM compared with cells cultured in CM (Figure 5C) and were not statistically different between these groups (Figure 5C). These data indicated that PAX9 did not affect the osteoblastic differentiation of iPDLSCs.

### 3.7. Adipocytic Differentiation of iNC-Unt, iNC-siCont, and iNC-siPAX9

**Figure 6:**
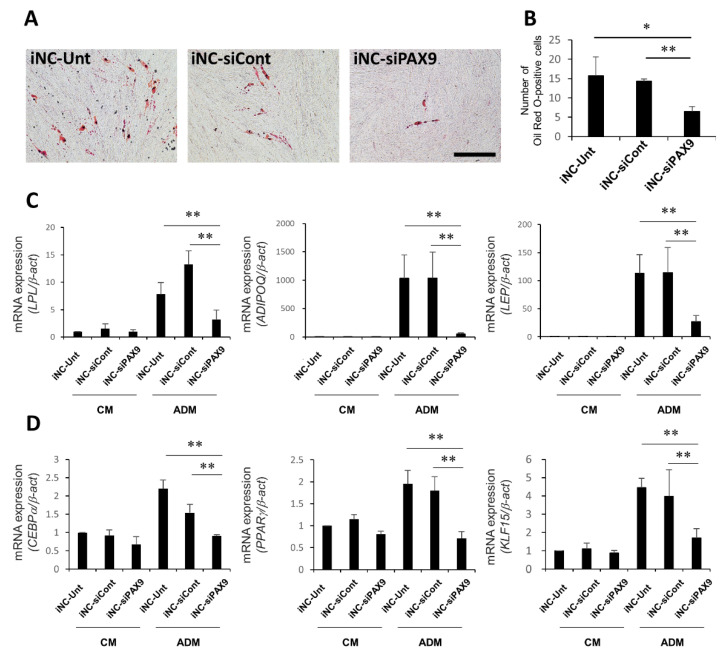
Limited adipocytic differentiation potential in iNC-siPAX9. (**A**) Representative Oil Red O-staining in iNC-Unt, iNC-siCont, and iNC-siPAX9 after 4 weeks of culture in ADM. Bar = 200 µm. (**B**) Numbers of Oil Red O-positive cells in iNC-Unt, iNC-siCont, and iNC-siPAX9 cultured in ADM for 4 weeks. (**C**,**D**) Quantitative real time RT-PCR analysis of adipocyte marker genes *LPL*, *ADIPOQ*, and *LEP* (**C**) and adipogenesis regulator genes *CEBPα*, *PPARγ*, and *KLF15* (**D**) in iNC-Unt, iNC-siCont, and iNC-siPAX9 after 4 weeks of culture in CM or ADM. *β-act* was used as an internal standard. Data are portrayed as the mean ± standard deviation (*n* = 4). * *p* < 0.05 ** *p* < 0.01. ADM, adipocytic differentiation medium; CM, conditioned medium; iNC, induced pluripotent stem cell-derived neural crest-like cells.

After 4 weeks of culture in ADM, iNC-Unt and iNC-siCont formed large number of lipid droplets, while iNC-siPAX9 generated only a small number of lipid droplets (Figure 6A). Indeed, there were statistically significant differences in numbers of cells exhibiting lipid droplets between iNC-Unt and iNC-siPAX9, and iNC-siCont and iNC-siPAX9 (Figure 6B). In contrast, no droplets were observed in cells treated with CM (Figure S1B(I–III)). Expression of adipocyte-related markers *LPL*, *ADIPOQ*, and *LEP* was upregulated in iNC-Unt, iNC-siCont, and iNC-siPAX9 cultured in ADM compared with cells cultured in CM (Figure 6C). Moreover, their expression was downregulated in iNC-siPAX9 cultured in ADM compared with iNC-Unt and iNC-siCont cultured in ADM (Figure 6C). Expression of regulator genes for adipogenesis (*CEBPα*, *PPARγ*, and *KLF15*) was also suppressed in iNC-siPAX9 cultured in ADM relative to iNC-Unt and iNC-siCont cultured in ADM (Figure 6D). These data indicated that PAX9 was involved in the regulation of adipocytic differentiation in iPDLSCs.

## 4. Discussion

iNCs require culture on ECM derived from human PDL cells to differentiate into iPDLSCs [16]. It has been reported that the ECM is involved in determining the fate of stem cells such as epidermal stem cells, neural stem cells, and bone marrow stem cells [42,43,44]. Therefore, human PDL cell-derived ECM plays important roles in the generation of iPDLSCs. The morphology, iPDLSC-associated gene expression, and mesenchymal lineage cell differentiation of iNCs cultured on ECM derived from HPDLC-3S varied from cells cultured on ECM derived from HPDLC-3U, although both HPDLC-3S and HPDLC-3U were human PDL cells. HPDLCs were isolated from different individuals (HPDLC-3S: 23-year-old male, HPDLC-3U: 25-year-old female) and some factors, such as sex and age, can reportedly affect the composition of ECM [23]. Recent studies have demonstrated that mechanical stress regulates the ECM expression [45,46]. PDL tissue is located around tooth roots, therefore it continues to receive the occlusal force. Additionally, there are individual differences in the occlusal force [47]. Therefore, it was suggested that there are individual differences in the ECM components in the periodontal ligament tissues exposed to mechanical loading. CAGE results also demonstrated that gene expression patterns of HPDLC-3S and HPDLC-3U were different to some extent; specifically, 693 genes were upregulated and 675 genes were downregulated in HPDLC-3U by >2-fold relative to HPDLC-3S. These results indicate that differences in the composition of ECM between HPDLC-3S and HPDLC-3U likely cause of the observed difference in their ability to induce iPDLSCs from iNCs.

Our previous study demonstrated that iNCs cultured with the major components of ECM in human PDL cells, COL1 and POSTN, displayed lower expression of iPDLSC-associated genes relative to iPDLSCs. Moreover, a previous report revealed that artificially isolated single ECM components were insufficient to mimic the complex structure and complete function of natural ECM [48]. Therefore, we aimed to identify the transcription factor regulating the production of ECM in human PDL cells, which is responsible for the induction of iNCs to iPDLSCs. We focused on the transcription factor *PAX9* based on results from CAGE and GSEA technologies. PAX9 belongs to the paired box family that encodes a group of growth- and development-regulation-related transcription factors [49]. GSEA results also indicated the involvement of *PAX9* in head morphogenesis, body morphogenesis, face development, tissue development, and regulation of animal organ morphogenesis. PAX9 expression was previously confirmed in various tissues such as thymus, parathyroid, tonsil, vagina, and cervix [50,51]. Its expression was also identified in oral tissues including salivary glands, taste papilla of the tongue, lip, and developing palatal shelves [52]. Moreover, Pax9 is widely expressed in dental mesenchyme of developing tooth germ, whereby defects in Pax9 were associated with a lack of tooth buds and hypodontia [53]. Dental mesenchyme cells, which originate from neural crest cells, produce PDLSCs that ultimately form PDL tissue [3]; therefore, PAX9 is suggested to be a crucial factor regulating the differentiation of neural crest cells into PDLSCs. 

PAX9 reportedly regulates some ECM genes; for example, exogenous *PAX9* increases expression of dentin matrix protein 1 in human iNCs [54], while endogenous Pax9 positively regulates *Col2a1* and *Acan* in mouse prechondrogenic mesenchymal cells of the intervertebral discs [55]. Additionally, several studies demonstrated the effects of ECM on multipotency of stem cells. Kearns et al. suggested that the ECM environment of the central nervous system is crucial for the maintenance of multipotency of neural stem cells [56], while Antoon et al. revealed that bladder-derived ECM was of great importance to maintain the multipotency in MSCs [57]. Interestingly, tendon stem/progenitor cells contacting ECM derived from the tendon of *Bgn^−/0^Fmod^−/−^* mice exhibited lower potential to differentiate into tenocytes compared with cells contacting ECM derived from wild-type mice [58]. These results strongly support our finding that ECM derived from 3U-siPAX9 impaired expression of iPDLSC-associated markers and the differentiation of adipocytes from iNCs. However, cell morphology, proliferation, surface marker expression, and osteoblastic differentiation did not vary between iNC-Unt3U, iNC-siCont, and iNC-siPAX9, suggesting that *PAX9* partially regulates ECM production in human PDL cells, although other transcription factors are likely involved. As with *PAX9*, *FOXF2* expression was significantly higher in HPDLC-3U compared with HDF and HPDLC-3S. GENE MANIA analysis demonstrated co-expression of *PAX9* with *FOXF2* (Figure S2A). FOXF2 is a specific mesenchymal transcription factor expressed in mesenchymal cells adjacent to the epithelium [59]. Ormestad et al. demonstrated that *Foxf2* promotes ECM synthesis in fibroblasts to support mouse gut development [60]. *Foxf2^−/−^* mice develop a cleft palate because of defects in ECM synthesis of the secondary palate [61]; intriguingly, this phenotype is consistent with that of *PAX9*-deficient mice exhibiting a cleft secondary palate at birth [52]. Collectively, these results indicate that FOXF2 may also be involved in the differentiation of iNCs into iPDLSCs via regulation of ECM synthesis in human PDL cells. However, the relationship between tooth development and FOXF2 was not well investigated; only one report demonstrated that the mutation of *FOXF2* was associated with the loss of early tooth markers [62]. Conversely, PAX9 was identified as a marker for prospective tooth mesenchyme in 1997 [63]. Following this report, various studies portrayed the involvement of PAX9 in tooth development. Based on these results, we considered that PAX9 would be more involved in tooth development than FOXF2 and we focused on *PAX9* in this study. Additionally, GENE MANIA analysis indicated physical interactions, co-expression, and co-localization of *PAX9* with transcription factor genes such as *MSX1*, *PAX2*, *PAX3*, *PAX4*, *PAX6*, and *PAX7* (Figure S2B). Among them, only the interaction between *PAX9* and *MSX1* has been clarified. Vieira et al. tested for possible *PAX9-MSX1* interactions by observing the transmission of marker alleles from parents, suggesting that *PAX9* interacts with *MSX* to cause tooth agenesis in humans [64]. Ogawa et al. further demonstrated that their interaction regulated *Bmp4* expression to determine the fate of the transition from bud stage to cap stage during tooth development [65]. CAGE results revealed slightly increased *MSX1* expression in HPDLC-3U relative to HDF (1.72-fold) and HPDLC-3S (1.12-fold). Therefore, further studies are essential to identify the interaction between *PAX9* and *MSX1*, and their involvement in regulation of ECM synthesis in PDL cells.

**Figure 7:**
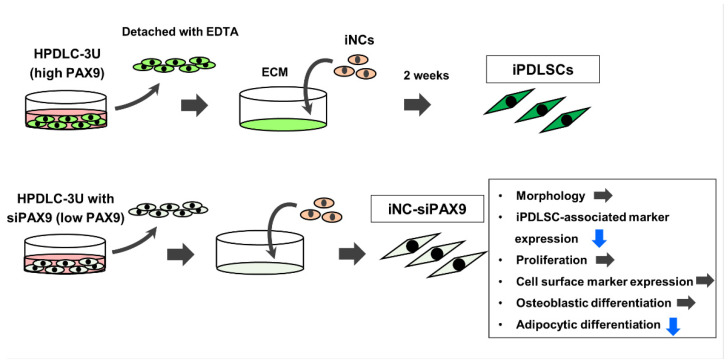
A schematic of the effects of PAX9 downregulation in HPDLC-3U HPDLC-3U cells highly express *PAX9*, while iNCs cultured on ECM derived from HPDLC-3U differentiate into iPDLSCs. siPAX9-transfected HPDLC-3U downregulates PAX9 gene expression. iNCs cultured on ECM derived from siPAX9-transfected HPDLC-3U decreased the expression of iPDLSC-associated markers and the ability to differentiate into adipocytes. ECM, extracellular matrix; iNC, induced pluripotent stem cell-derived neural crest-like cells.

In summary, we compared the ability of ECM derived from HDF, HPDLC-3S, and HPDLC-3U to induce differentiation of iNCs into iPDLSCs. iNCs cultured on ECM derived from HPDLC-3U displayed PDLSC-like phenotypes (Figure 7) and strong expression of *PAX9* compared with HDF and HPDLC-3S. siPAX9 transfection successfully downregulated PAX9 at gene levels in HPDLC-3U. iNCs cultured on ECM derived from siPAX9-transfected HPDLC-3U exhibited decreased expression of iPDLSC-associated markers and adipocyte differentiation ability (Figure 7). We report the involvement of *PAX9* in regulation of ECM production in human PDL cells, which plays important roles in the differentiation of iNCs into iPDLSCs. *PAX9* may be an effective marker for selecting human PDL cells that produce ECM responsible for the generation of iPDLSCs. Moreover, human PDL cells highly expressing *PAX9* may contribute to the progress of regenerative medicine for PDL tissues via efficient induction of iNCs to iPDLSCs by their ECM.

## 5. Conclusions

We report the involvement of PAX9 in regulation of ECM production in human PDL cells, which plays important roles in the differentiation of iNCs into iPDLSCs. PAX9 may be an effective marker for selecting human PDL cells that produce ECM responsible for the generation of iPDLSCs. Moreover, human PDL cells highly expressing PAX9 may contribute to the progress of regenerative medicine for PDL tissues via efficient induc-tion of iNCs to iPDLSCs by their ECM.

## Figures and Tables

**Table 1:** GenBank ID, primer sequences, product sizes, and annealing temperatures for quantitative reverse transcription polymerase chain reaction.

Target Gene (Abbreviation)	GenBank ID	Forward (Top) and Reverse (Bottom) Primer Sequences	Size of Amplified Products (bp)	Annealing Temperature (°C)
β-actin	NM_001101.5	5′-ATTGCCGACAGGATGCAGA-3′/ 5′-GAGTACTTGCGCTCAGGAGGA-3′	89	60
Alpha smooth muscle actin (aSMA)	NM_001613.4	5′-GACAATGGCTCTGGGCTCTGTA-3′/ 5′-CTGTGCTTCGTCACCCACGTA-3′	147	60
Osteoprotegerin (OPG)	NM_002546.4	5′-CTCGAAGGTGAGGTTAGCATGTC-3′/ 5′-TGGCACCAAAGTAAACGCAGAG-3′	196	60
Alkaline phosphatase (ALP)	NM_001177520.3	5′-GGACCATTCCCACGTCTTCAC-3′/ 5′-CCTTGTAGCCAGGCCCATTG-3′	137	60
Periostin (POSTN)	NM_006475.2	5′-CATTGATGGAGTGCCTGTGGA-3′/ 5′-CAATGAATTTGGTGACCTTGGTG-3′	167	60
Type1 collagen Alpha 1 (COL1A1)	NM_000088.3	5′-CCCGGGTTTCAGAGACAACTTC-3′/ 5′-TCCACATGCTTTATTCCAGCAATC-3′	148	60
Periodontal ligament-associated protein 1 (PLAP1)	NM_017680.5	5′-ATGGGAGTCTTGCTAACATACCAC-3′/ 5′-CAGAAGTCATTTACTCCCACTCTTG-3′	154	60
Forkhead box F2 (FOXF2)	NM_001452.2	5′-TCGCCTTACCTCAAGCAGC-3′/ 5′-AGAGTGATGCTGGTAACGGG-3′	165	60
SIX homeobox 2 (SIX2)	NM_016932.5	5′-GGCCGAGGCCAAGGAA-3′/ 5′-GGGCTGGATGATGAGTGGTC-3′	144	60
Distal-less homeobox 5 (DLX5)	NM_005221.6	5′-CAGAAGACTCAGTACCTCGCC-3′/ 5′-GTTACACGCCATTGGGTCG-3′	180	60
Paired box 9 (PAX9)	NM_001372076.1	5′-GCAGGAAGCCAAGTACGGT-3′/ 5′-TGTCACAGTTGTGGGGAGAC-3′	200	60
Transcription factor AP-4 (TFAP4)	NM_003223.3	5′-CACATCCCGGCAAAATCTGG-3′/ 5′-CCATGGCGTCACTGTCTGAG-3′	185	60
Osteocalcin (OCN)	NM_199173.4	5′-CCCAGGCGCTACCTGTATCAA-3′/ 5′-GGTCAGCCAACTCGTCACCAGTC-3′	112	60
Bone morphogenetic protein 2 (BMP2)	NM_001200.2	5′-TCCACTAATCATGCCATTGTTCAGA-3′/ 5′-GGGACACAGCATGCCTTAGGA-3′	73	60
Bone sialoprotein (BSP)	MN_004967.3	5′-CTGGCACAGGGTATACAGGGTTAG-3′/ 5′-ACTGGTGCCGTTTATGCCTTG-3′	181	60
Lipoprotein lipase (LPL)	NM_000237.2	5′-GACTCGTTCTCAGATGCCCT-3′/ 5′-ACTTCAGGCAGAGTGAATGGG-3′	145	60
Adiponectin (ADIPOQ)	NM_001177800.2	5′-CAGGAAACCACGACTCAAGGG-3′/ 5′-CCGGTTTCACCGATGTCTCC-3′	200	60
Leptin (LEP)	NM_000230.3	5′-GCTGTGCCCATCCAAAAAGTC-3′/ 5′-CCAGTGTCTGGTCCATCTTGG-3′	178	60
CCAAT enhancer binding protein alpha (CEBPa)	NM_004364.4	5′-GGTGGACAAGAACAGCAACGA-3′/ 5′-GTCATTGTCACTGGTCAGCTC-3′	136	60
Proliferator-activated receptor gamma (PPARg)	NM_138712.5	5′-TATTCTCAGTGGAGACCGCC-3′/ 5′-TGAGGACTCAGGGTGGTTCA-3′	115	60
Kruppel like factor 15 (KLF15)	NM_014079.4	5′-TACACCAAAAGCAGCCACCTC-3′/ 5′-CTGGTACGGCTTCACACCTG-3′	153	60

## Data Availability

CASE analysis data generated and/or analyzed during the current study are available in the Gene Expression Omnibus (GEO) repository, Accession Number GSE208250. The other datasets either generated and analyzed or just analyzed in this study are available from the corresponding author upon reasonable request.

## Supplementary Materials

The following supporting information can be downloaded at: https://www.mdpi.com/article/10.3390/biomedicines10102366/s1, Figure S1: Culturing iNC-Unt, iNC-siCont, and iNC-siPAX9 in control medium; Figure S2: GENE MANIA for *FOXF2*, *SIX2*, *DLX5*, *PAX9*, and *TFAP4*.
