# Supplementary material for: PAX9 Is Involved in Periodontal Ligament Stem Cell-like Differentiation of Human-Induced Pluripotent Stem Cells by Regulating Extracellular Matrix
Source: Biomedicines. 2022 Sep 22;10(10):2366. doi: 10.3390/biomedicines10102366 (PMC9598762; doi:10.3390/biomedicines10102366)
Supplement: Supplementary file 1 [file biomedicines-10-02366-s001.zip › biomedicines-1905179-supplementary.pdf]

Figure S1.

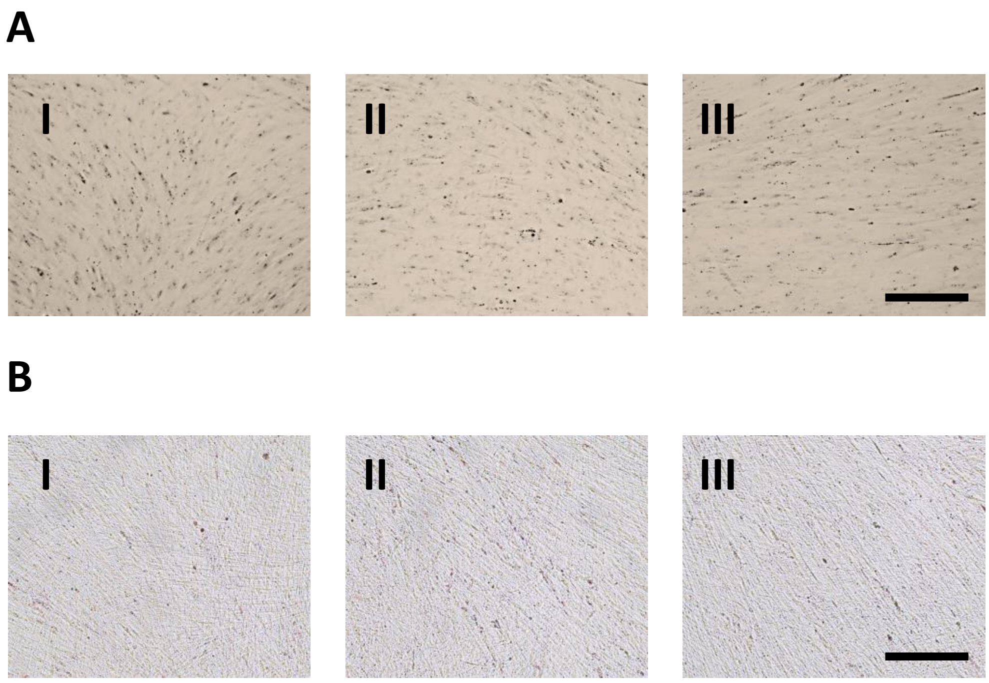

Figure S1. Culturing iNC-Unt, iNC-siCont, and iNC-siPAX9 in control medium

A: Representative Alizarin Red S-staining in iNC-Unt (I), iNC-siCont (II), and iNC-siPAX9 (III) after 3 weeks of culture in CM. Bar = 100 μm. B: Representative Oil Red O-staining in iNC-Unt (I), iNC-siCont (II), and iNC-siPAX9 (III) after 4 weeks of culture in CM. Bar = 100 μm. CM, conditioned medium; iNC, induced pluripotent stem cell-derived neural crest-like cells.

Figure S2.

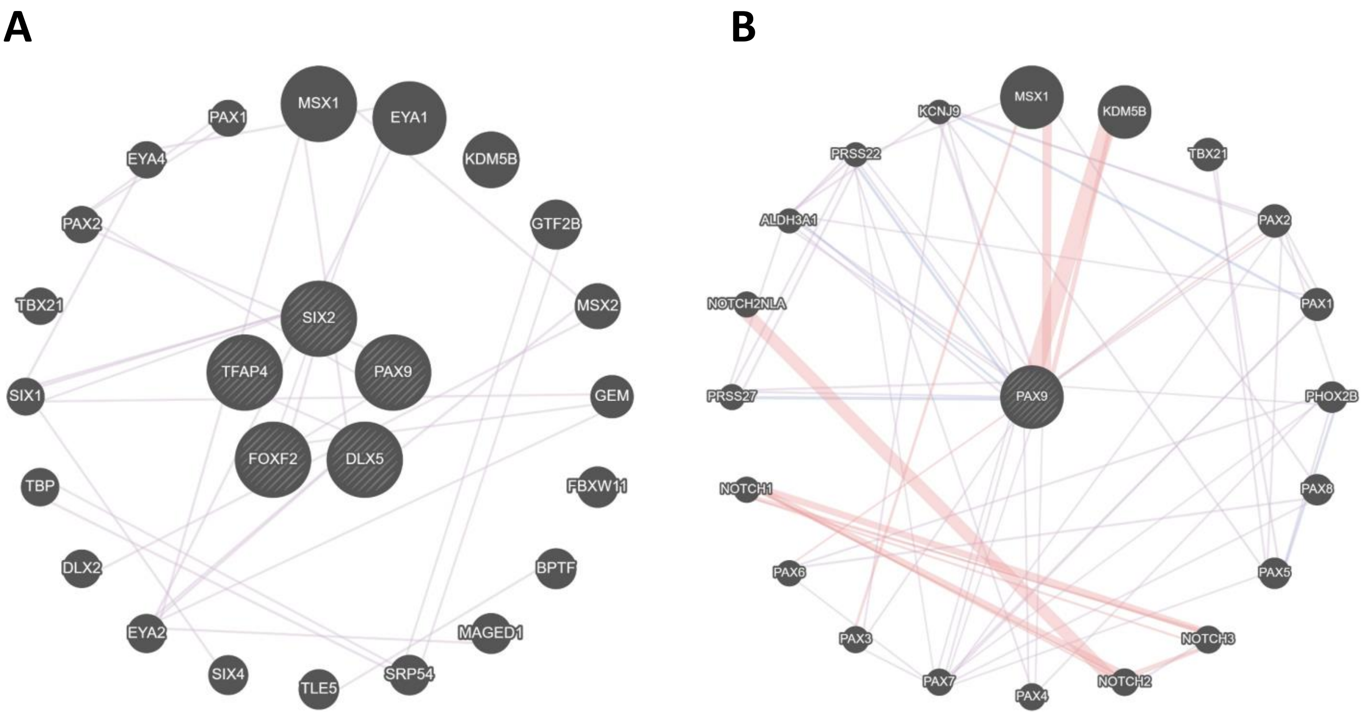

Figure S2. GENE MANIA for *FOXF2*, *SIX2*, *DLX5*, *PAX9*, and *TFAP4*

A: GENE MANIA result for co-expression of *FOXF2*, *SIX2*, *DLX5*, *PAX9*, and *TFAP4*. Purple lines indicate co-expression of genes. B: GENE MANIA result for physical interactions, co-expression, and co-localization in *PAX9*. Orange, purple, and blue lines indicate physical interactions, co-expression, and co-localization, respectively.
